# Analysis and prediction of interactions between transmembrane and non-transmembrane proteins

**DOI:** 10.1186/s12864-024-10251-z

**Published:** 2024-04-24

**Authors:** Chang Lu, Jiuhong Jiang, Qiufen Chen, Huanhuan Liu, Xingda Ju, Han Wang

**Affiliations:** School of Psychology, School of Information Science and Technology, Institute of Computational Biology, Northeast Normal University, Changchun, China

**Keywords:** Transmembrane protein, Protein-protein interaction, Convolutional neural network, Enrichment analysis, Subcellular locations

## Abstract

**Background:**

Most of the important biological mechanisms and functions of transmembrane proteins (TMPs) are realized through their interactions with non-transmembrane proteins(nonTMPs). The interactions between TMPs and nonTMPs in cells play vital roles in intracellular signaling, energy metabolism, investigating membrane-crossing mechanisms, correlations between disease and drugs.

**Results:**

Despite the importance of TMP-nonTMP interactions, the study of them remains in the wet experimental stage, lacking specific and comprehensive studies in the field of bioinformatics. To fill this gap, we performed a comprehensive statistical analysis of known TMP-nonTMP interactions and constructed a deep learning-based predictor to identify potential interactions. The statistical analysis describes known TMP-nonTMP interactions from various perspectives, such as distributions of species and protein families, enrichment of GO and KEGG pathways, as well as hub proteins and subnetwork modules in the PPI network. The predictor implemented by an end-to-end deep learning model can identify potential interactions from protein primary sequence information. The experimental results over the independent validation demonstrated considerable prediction performance with an MCC of 0.541.

**Conclusions:**

To our knowledge, we were the first to focus on TMP-nonTMP interactions. We comprehensively analyzed them using bioinformatics methods and predicted them via deep learning-based solely on their sequence. This research completes a key link in the protein network, benefits the understanding of protein functions, and helps in pathogenesis studies of diseases and associated drug development.

**Supplementary Information:**

The online version contains supplementary material available at 10.1186/s12864-024-10251-z.

## Background

Protein-protein interactions (PPIs) provide a systematic point of view for understanding the life process including DNA replication, protein modification, and signal transduction [1, 2]. The interactions between transmembrane proteins and non-transmembrane proteins (TMP-nonTMP interaction) are a special kind of PPIs that realize intracellular and extracellular signaling, regulate energy metabolism, and many other functions throughout the cell life cycle [3]. The pathogenesis of many serious diseases associated with TMPs, such as Alzheimer's disease [4–6], Parkinson's disease [7, 8], Metabolic abnormalities [9], immune system diseases [10], and many other kinds of diseases. Since TMPs are major drug targets, TMP-nonTMP interactions directly affect drug metabolism and usually occupy the position of hub nodes in related pathways [11]. The study of TMP-nonTMP Interactions will promisingly contribute to the understanding of protein functions, completing the PPI network, exploring the pathogenesis of diseases, and discovering the potential drug targets [3, 11, 12].

Biological experiments are the most reliable approach to determinate molecular interactions that provide accurate PPIs [13, 14]. Popular experimental methods for PPIs are the yeast-two-hybrid (Y2H) system [15], affinity purification followed by mass spectrometry (AP-MS) [16], and literature-derived low-throughput experiments [17]. Y2H is a powerful method to detect PPIs occurring in the nucleus but is not suitable for detecting TMP-TMP or TMP-nonTMP interactions. Influenced by the membrane, TMPs differ greatly from water-soluble proteins in terms of microenvironment, structure, and functions, resulting in different docking locations and mechanisms with molecules (including ligands and proteins) [14, 18, 19]. The split-ubiquitin system provides a method for examining the interactions of membrane proteins in their native environment [20]. In 2014, Petschnigg et al. developed the mammalian-membrane two-hybrid assay (MaMTH), a split-ubiquitin-based two-hybrid system developed to assess PPIs of membrane proteins [21]. In 2017, Saraon et al detected the integral membrane PPIs in the context of living mammalian cells [22]. With the development of experimental techniques, more and more TMP-TMP and TMP-nonTMP interactions have been detected [23–25]. However, these experimental techniques are labor-intensive and time-consuming. The amphipathic structure makes it complicated to determine the interactions between TMPs and nonTMPs through biological experiments on a large scale [26]. When performing transduce signals, TMP PPIs are transient where protein partners associate and dissociate temporally. It is difficult to detect those kinds of PPIs since they are less likely to be colocalized [17]. Furthermore, the detection results are frequently observed in high ratios of false positives and false negatives [27]. To overcome these disadvantages, computational models can provide auxiliary validation and predict new PPIs.

Computational methods enable the screening of large-scale molecular interactions and are effective adjunct strategies for biological experiments. Since both sides of most known PPIs are water-soluble proteins, many PPI prediction models simply exclude or ignore TMP-associated interactions (TMP and water-soluble protein are not distinguished) [28]. Although these models were not developed for TMP-nonTMP interactions, the impressive works facilitate the development of molecular interaction prediction and enlighten our work. Computational methods for predicting PPIs can be divided into sequence-based, structure-based, and template-based methods. Sequence-based algorithms only apply the primary sequence of proteins as input, without the secondary or tertiary structure information obtained experimentally. Compared with structural information, protein primary sequence is more accurate, stable, and easier to obtain. Theoretically, the primary sequence of a protein contains all the information about its structure and function, and sequence-based predictors have been proved to achieve great performance [29, 30]. According to the algorithm, PPI prediction methods can be divided into traditional machine learning-based methods and deep learning-based methods. Shen et al. provide a Conjoint Triad (CT) method to describe protein sequence for predicting PPIs with SVM [31]. LDA-RF obtains low dimensional latent topic features from protein sequences and then adopts the scalable random forest to predict human PPIs [32]. iPPI-PseAAC (CGR) incorporates the information of “chaos game representation” into the Pseudo Amino Acid Composition (PseAAC) and then adopts a random forest to classify PPIs [33]. GTB-PPI predicts PPIs based on Gradient Tree Boosting (GTB) by fusing PseAAC, pseudo-position-specific scoring matrix (PsePSSM), reduced sequence, and index-vectors (RSIV), and autocorrelation descriptor (AD) [2]. Those methods rely on a large number of manual features like Position Specific Score Matrix (PSSM) profiles, domain information, and predicted secondary structures. Those features require plenty of expert knowledge and redundant data processes [34]. The characteristics of deep learning algorithms determine that they can abandon complicated feature engineering, but directly make more accurate predictions based on original information. Sun et al. combined Stacked AutoEncoder (SAE) with protein sequence to predict PPIs [35]. Zhang et al. used the DNNs model that takes Auto Covariance (AC) descriptor as the input to predict PPIs [36]. Li et al designed a CNN and LSTM-based deep learning model to predict PPIs from one-hot encoding [37]. DNN-PPI used an Auto Covariance (AC) descriptor and a Conjoint Triad (CT) descriptor for the prediction of PPI [38]. Wang et al. embed amino acids in diverse vector spaces to predict PPIs [39]. PIPR, an end-to-end framework that embeds sequence by the vector obtained from a pre-trained model, relieves the data pre-processing efforts to predict PPIs and obtains the start of art result [40].

The above prediction models have achieved considerable results in the prediction of water-soluble protein interactions, but there are great particularities in the TMP-related dockings. The dockings of TMPs with other molecules are more complex: they may occur on lipid-soluble surfaces in transmembrane regions, water-soluble surfaces in non-transmembrane regions, interfaces on membrane surfaces, and channels within TMPs. This poses a great challenge to TMP-molecular interaction prediction, and modeling based on molecular types is a feasible solution. Some studies have been started to pay attention to probing TMP-TMP interactions. Duart et al made a systematic review of methodological approaches for the analysis of transmembrane domain interactions [41]. Khazen et al proposed PPIMem, a novel approach for predicting transmembrane protein-protein complexes [28]. The important biological significance of TMP-nonTMP interaction cannot be ignored. However, the research on them is still limited in the wet experimental stage, lacking analysis and modeling from computational perspectives.

In this study, we firstly performed statistical analyses of the known TMP-nonTMP interactions from different perspectives: a) The distribution of species, protein families, and subcellular locations were calculated; b) The enrichment items of Gene Ontology (GO) and Kyoto Encyclopedia of Genes and GenomesKEGG pathway were analyzed, c) The TMP-nonTMP interaction network was constructed, the hub proteins and critical sub-networks models of the network were found. After the comprehensive analysis, We proposed an end-to-end prediction model to identify potential TMP-nonTMP interaction, which is convenient and efficient. Within our framework, two proteins in an interacting pair were connected head to tail and encoded by a one-hot code. Then, a CNN model was applied to extract features from sequence pairs automatically and fed into a fully connected layer for sorting. The experimental results over the independent validation demonstrated considerable prediction performance with an MCC of 0.541. This research completes a key link in the PPI network and is beneficial for exploring the drug target. Materials and code related are available at https://github.com/NENUBioCompute/SeqTMPPI/.

## Methods

### Benchmark datasets

We used the TMP-nonTMP interactions recorded in the IntAct [42] as the positive samples. After constructing negative samples, removing similar protein pair sequences and irregulated proteins, we obtained 64,939 positive samples and 64,939 negative samples to build our benchmark datasets. To optimize a model, we built *MINI* to explore the best composition of parameters.

Protein annotations were extracted from UniProt [43], including keywords, subcellular location, species, and so on. TMPs are annotated with ‘KW-0812’ in the keywords field while nonTMPs are not. With the suppose that proteins from different subcellular locations do not interact with each other [32], we randomly composed TMPs and nonTMPs in UniProt/SwissProt as negative protein samples. Pairs with proteins annotated with the same subcellular location terms were removed. Protein sequences consisting of < 50 or > 2,000 (details illustrated in Additional file 1) amino acid residues, or containing unknown residues were removed. Pairs showing pair-wise sequence identity ≥ 40% via CD-HIT algorithm [44] were removed. Details are as follows: (1) We put sequence information of TMPs and nonTMPs in all the samples (positive samples and negative samples) to CD-HIT tools [44]. (2) proteins were clustered in a group if their amino acid residues showed sequence identity ≥ 40% via the CD-HIT algorithm. (3) Check any two protein pairs A-B, A’-B’. If A and A’ are in the same cluster (sequence identity ≥ 40%), meanwhile B and B’ are in the same clusters, we deleted A’-B’.

After pretreatment, we collected a total number of 64,939 TMP-nonTMP pairs as positive samples (*POSI*) and 84,726 negative samples. *POSI* was used in statistical analysis to investigate the mechanisms of TMP-nonTMP interactions.

A balanced dataset will be beneficial to train a deep learning model. To build a balanced dataset, we mixed 64,939 positive samples and 64,939 negative samples (*NEGA*) to get a balanced dataset (*BENCH*) and then divided them into 5 subsets. Each subset was divided into a training set, validating set, and independent testing set according to the ratio of 8:1:1. Detailed statistic of the samples in *BENCH* was illustrated in Additional file 1. To avoid contingency, we trained and tested the model with each group of datasets separately, using the average value of 5 experiments as the final performance.

We construct a small dataset (*MINI*) to explore the best composition of parameters. We collected a total number of 2,049 TMP-nonTMP pairs as positive samples from the IMEx Consortium mutations data set (released on May 2, 2019). Then, we obtained 2,049 negative samples by randomly pairing the TMP and nonTMP in Swiss-Prot (released on Jan 9, 2020). All the data processes of *MINI* are the same as BENCH except for removing the pairs showing pair-wise sequence identity≥ 40% because *MINI* did not have enough scale to eliminate redundancy. To avoid the contingency of negative sample selection, we repeated the above processes 5 times to form 5 datasets. We trained and tested the model with each dataset separately that all the experimental results in this paper were the average value of 5 experiments.

### Statistical analysis

We analyzed the protein in *POSI* according to their annotations. Their annotations were extracted from UniProt [43], including protein family, subcellular location, species, and so on. Gene Ontology (GO) [45] annotations and pathway annotations were extracted from the GO database and KEGG PATHWAY database.

### Protein families

The Protein family (Pfam) [46] is a collection of related protein regions, providing insights into protein function. Functional regions are termed domains and in nature, proteins are diverse with various combinations of them. Proteins in the same family also share a common evolutionary, reflected by their related functions and similarities in sequence or structure. Furthermore, protein families are often arranged into hierarchies, with proteins that share a common ancestor subdivided into smaller, more closely related groups. The Pfam database (http://pfam.xfam.org/, version 34.0) [47] annotates proteins with protein families information, which is referenced in UniProt. In this paper, Pfam information of each protein was extracted from the UniProt field ‘dbReference’, the type of this field attribute was set as ‘Pfam’. All the protein family appears in this field were collected for analysis.

### Enrichment analysis

Enrichment analysis was done to compare the genes in TMP-nonTMP interactions with annotated gene sets in the GO and Encyclopedia of Gene and Genome (KEGG) [48] http://www.kegg.jp/, aiming to obtain biological information. Several databases are managed by KEGG, among them, genes in KEGG GENE were used as background genes in the enrichment analysis procedure. And GO items annotations and pathway annotations of query genes were extracted from it. All the GO items were defined in the GO database and pathways were defined in KEGG PATHWAY.

GO enrichment analysis is a very important bioinformatics analysis, through which researchers can observe the enrichment of genes encoding of TMPs or nonTMPs, and make out gene products in molecular function (MC), biological process (BP), and Cellular Component (CC) of GO enriched terms. KEGG Pathways provide a systematic way to understand the functions of individual genes and proteins that contribute to normal physiology and disease [49], each enriched item means a pathway in KEGG PATHWAY database. *P*-value is used to measure the enrichment of each type of GO. When the *p*-value is less than 0.05, this term is considered to have statistical significance. However, the *P*-value requires proper adjustment since the probability of committing false statistical inferences would considerably increase when more than one hypothesis is simultaneously tested. We use the P.adjust, calculated by Benjamini-Hochberg (BH) adjustment algorithm [50], to adjust the origin *P*-value.

Profiler R package [51] was used to perform GO enrichment analysis and KEGG pathway enrichment analysis. Firstly, we mapped the TMPs and nonTMPs in *POSI* to the gene list by UniProt annotations. Then took this gene list as input and set the cut-off criterion as an adjusted *P*-value <0.05, background gene list is from the KEGG GENE database. Finally, we obtained enrichment results separately for the genes of TMPs and nonTMPs. The significantly go enriched terms for biological process (BP), cellular component (CC), and molecular function (MF) were further ranked by *p*-value and visualized. Each category contains 10 analysis terms with the smallest *P*-value. A similar procedure was performed for KEGG pathway analysis with the same background gene list as GO enrichment analysis.

### Predictor construction

#### Encoding of protein pair

The amino acids in the protein sequence need to be encoded as numbers since deep learning models can interpret only numeric data. In this work, One-Hot [52–54] strategy was adopted to encode amino acids in the protein sequence. After that, an M $\times$ N binary matrix was obtained, where the M equals the max protein sequence length of 2,000 (cover more than 99% of sequences in UniProt) and the N equals the number of amino acid types 21 (20 for the natural amino acids types and one for the padding mark as a special type). Each amino acid was represented as a 1 $\times$ 21 vector: one the element corresponding to itself while zero for the remaining elements. Finally, concatenated the N-segment of nonTMP linearly to the C-terminal of TMP, a matric of 4,000 $\times$ 21 was obtained.

#### Model details

**Fig. 1:**
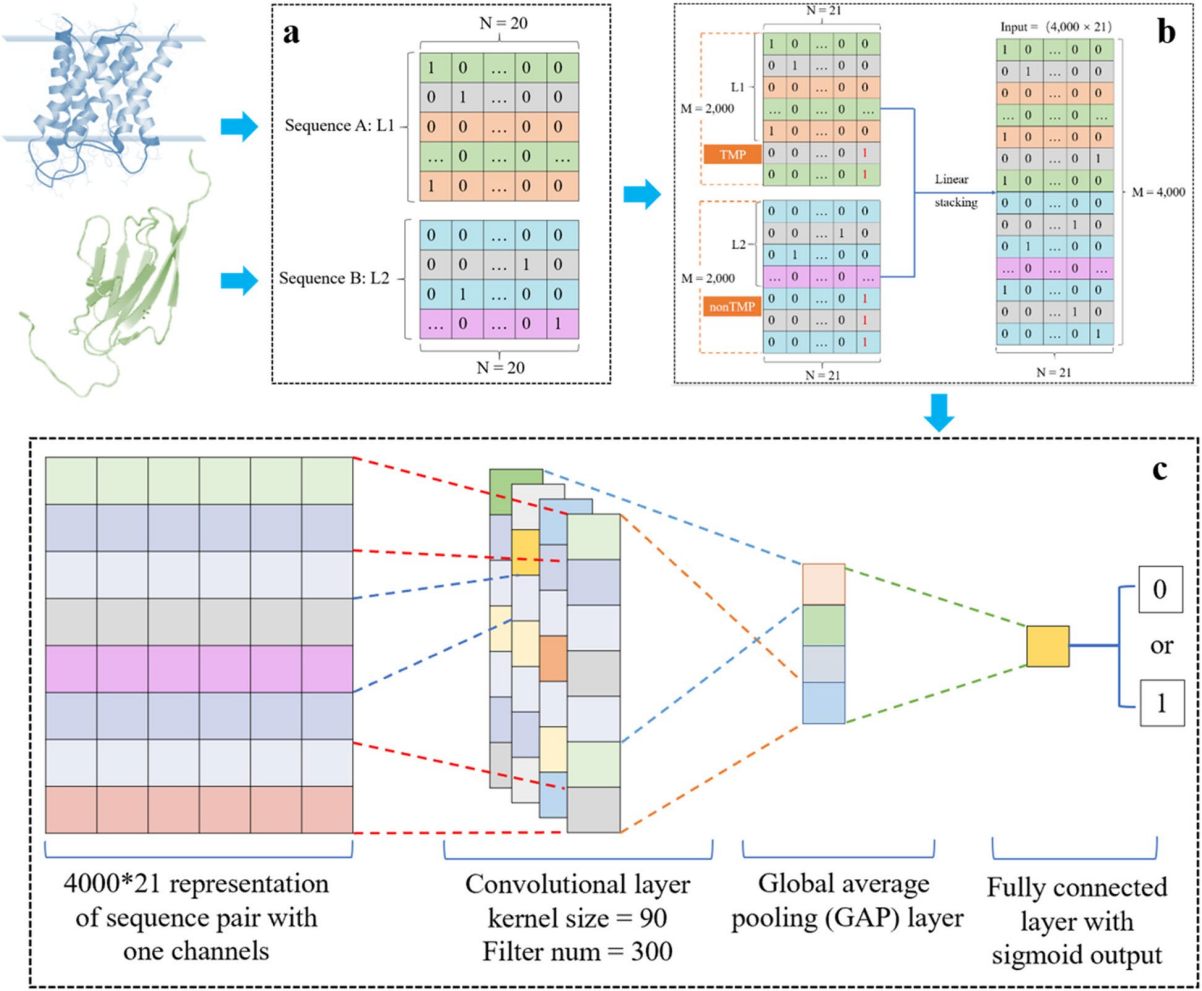
Workflow of SeqTMPPI. **a** Sequence of the transmembrane protein and non-transmembrane protein. **b** The first step is to pad each protein sequence to the same length (2,000), then, encode the protein sequence as a vector by the One-Hot strategy and concatenate the proteins in a pair linearly. **c** Finally, a CNN model with a GAP layer was applied to learn the pair-wise pattern of the concatenated sequence to predict interactions betweenTMP and nonTMP

Deep learning models can learn advanced abstract features from raw inputs, showing a good performance by reducing the noise effect embedded in the original features. The convolutional neural network (CNN) [55], typical architecture of deep learning, has been used in biology for protein prediction recently [39, 56], proving that CNN can be successfully applied in a sequence problem. Proposed in 2014, Global Average Pooling (GAP) [57] replaces the traditional fully connected layers in CNN and is widely used for sequential work. Here, we applied a one-layer CNN architecture for our machine learning classifier. As illustrated in Fig. 1, we padded each protein sequence to the same length (2,000), encoded the protein sequence as a vector by the One-Hot strategy, concatenated the proteins in a pair linearly, then sequentially added a CNN layer as the input layer, a GPA as the hidden layer, a fully connected layer as the output layer.

All the methods were developed in the Python3.6 program language. Using TensorFlow [58] as a backend, deep learning algorithms were implemented by Keras [59], Scikit-learn [60] libraries of python were used for evaluating algorithms. For all protein sequences, the model input the same shape of a matrix, which have been elaborated in section Encoding of Protein Pair.

According to the tuning and exploring in the model (details illustrated in Additional file 1), we settled the hyper-params and dataset for our model. Final hyper-params for kernel size, filters number, and batch size is 90, 300, 90 separately. In this work, we randomly divided the benchmark data set (*BENCH*) into five subsets (details illustrated in Additional file 1). Each subset was divided into a training set, validating set, and independent testing set according to the ratio of 8:1:1.After tuning the params, we set the kernel size as 90, filter num as 300, and batch size as 70. That means, for each complete training, 70 pieces of data (length is 4,000 and channel is 21) were fed into the model. With 300 filters in which the kernel size is 90, the CNN layer extracted a series of feature maps. Function ‘Rectified Linear Unit (ReLU)’ was applied in this layer. The GAP layers calculate the average of all the feature maps and pass the result into the output layer, we applied a ‘Sigmoid’ function for getting the classification result. Finally, iterated the procedure 80 times. The early stop strategy was used to end the training procedure ahead when the absolute change of the loss value was < 0.0003.

## Results

### Species distribution

**Fig. 2:**
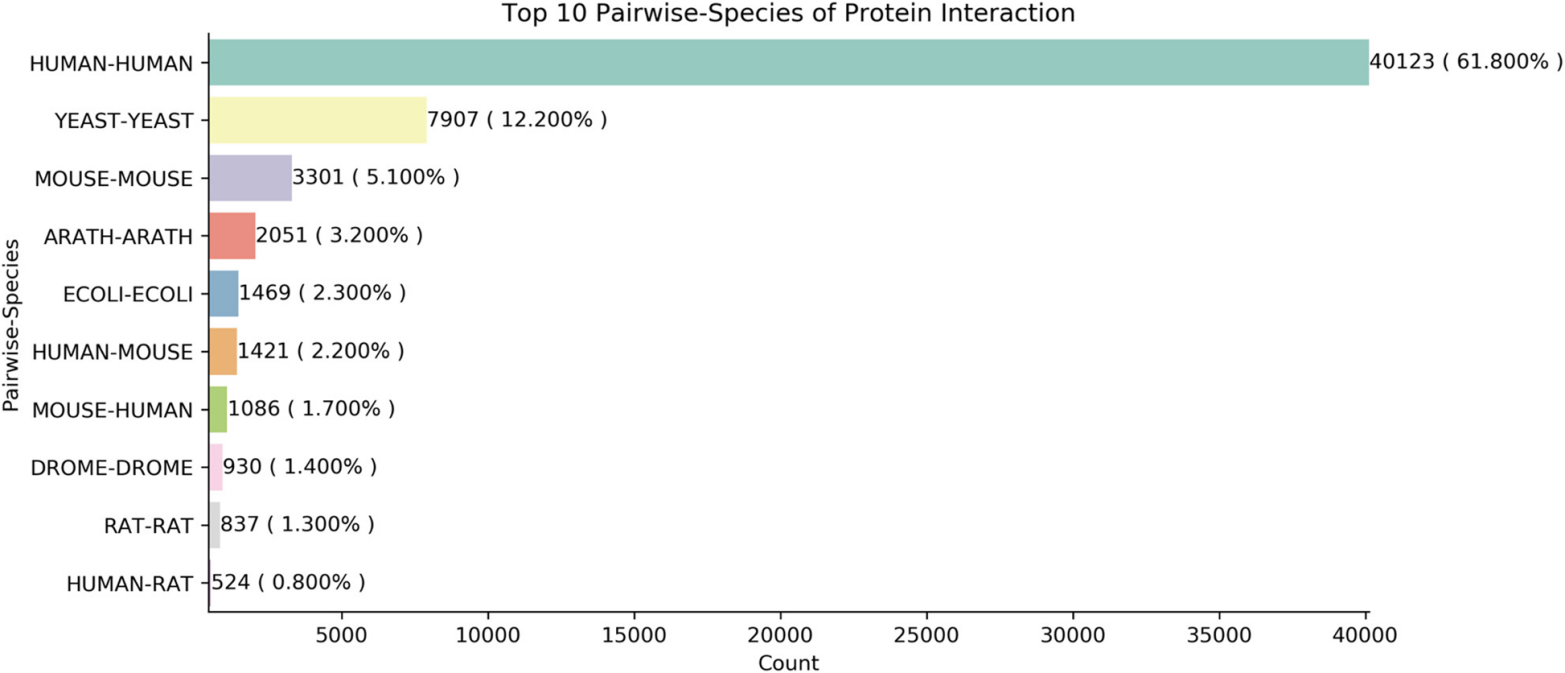
Distribution of the top 10 pairwise species of protein interactions. In this figure, the vertical axis represents the top ten species pairs, and the horizontal axis represents the number of interactions contained in each pairwise species. The top three of them are intraspecific interactions of humans, yeast, and mouse, accounting respectively for 61.800%, 12.200%, and 5.100% of all the situations

We counted the frequency of interactions when both participants of the interactions belong to the same species. As is shown in Fig. 2, the top 10 of them are human-human, yeast-yeast, mouse-mouse, arath-arath (Arabidopsis thaliana), ecoli-ecoli (Escherichia coli), human-mouse, mouse-human, drome-drome (Drosophila melanogaster), rat-rat, human-rat. Among them, TMP-nonTMP interactions of human-human occupied a big part, which means our datasets contain numerous human protein interaction patterns.

### Distribution of protein families

**Fig. 3:**
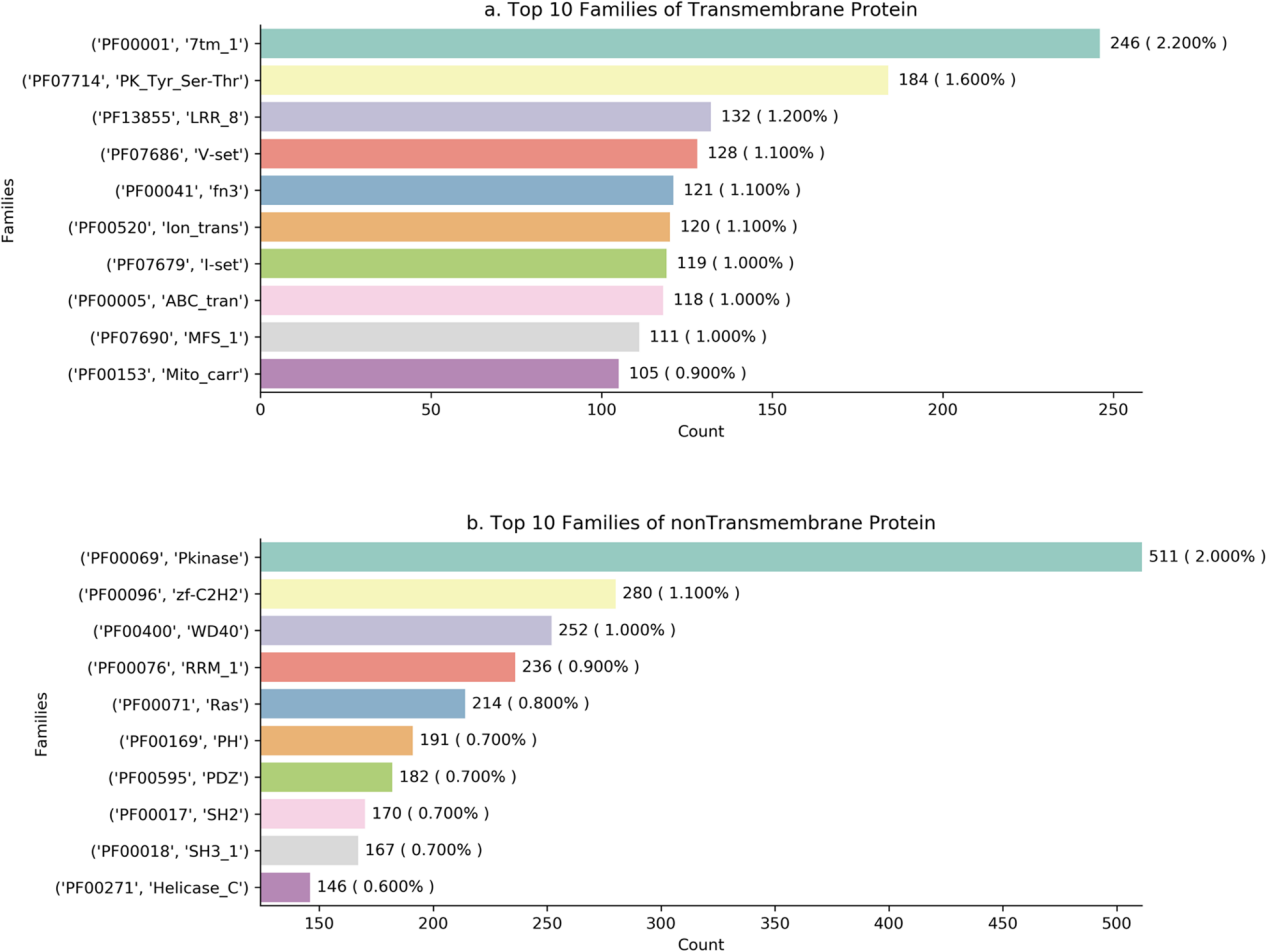
Distribution of the top 10 protein families. In this figure, the vertical axis represents the top ten protein family types, and the horizontal axis represents the number of proteins contained in each protein family. The top three protein families of transmembrane protein are 7tm_1, PK_Tyr_Ser-Thr, LRR_8, accounting respectively for 2.200%, 1.600%, and 1.200% of the transmembrane proteins; the top three protein families of non-transmembrane protein distribution are Pkinase, zf-C2H2, WD40, and accounting respectively for 2.000%, 1.100%, and 1.000% of the non-transmembrane proteins

Interaction between two proteins is a special function in biology. As is illustrated in the section Protein Families, protein functions can be inferred by the protein family. Here, By analyzing the statistical distribution of families of proteins, we investigated which families are the interactions between TMP (Fig. 3a) and nonTMP (Fig. 3b) closely related. Figure 3 shows the top 10 frequently occurring items for protein family in *POSI*.

### Subcellular locations of the proteins

**Fig. 4:**
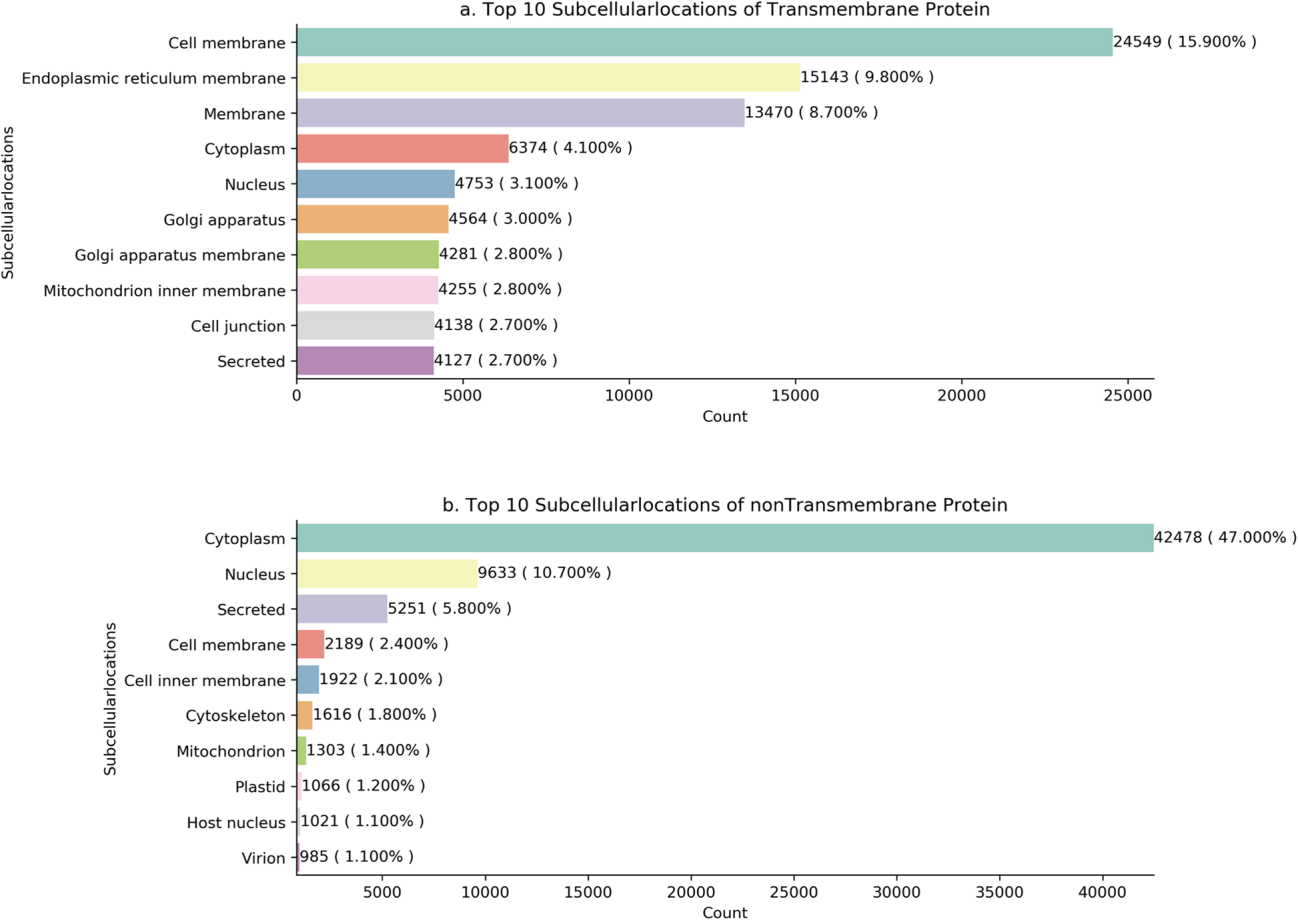
Distribution of the top 10 subcellular locations of the proteins. In this figure, the vertical axis represents the subcellular locations of proteins, and the horizontal axis represents the number of proteins contained in each subcellular location. **a** The top 10 subcellular locations of the TMPs. **b** The top 10 subcellular locations of the nonTMPs

To understand where TMP-nonTMP interactions often occur, we counted the subcellular locations of the TMPs since the nonTMPs can freely move. Figure 4a shows the top 10 subcellular locations of the TMPs are the cell membrane, endoplasmic reticulum membrane, cytoplasm, nucleus, golgi apparatus, and related membrane, mitochondrion inner membrane, cell junction, secreted. Most interactions are taking place at the cell boundary and they perform vital functions to transform information between environment and cell. Among them, cell membrane, endoplasmic reticulum membrane, cytoplasm accounting respectively for 15.900%, 9.800%, and 8.700% of all the transmembrane protein subcellular locations.

Furthermore, to explore where the signal was coming from and where it was going, we counted the subcellular locations of the nonTMPs that could carry the signal and move around. Figure 4b shows that the top 10 subcellular locations of the nonTMPs are cytoplasm, nucleus, secreted, cell membrane, cell inner membrane, cytoskeleton, mitochondrion, plastid, host nucleus, virion. Most of the signals are stay in the cytoplasm while some of them are transferred into the nucleus or secreted outside the environment. Among them, cytoplasm, nucleus, secreted account respectively for 47.000%, 10.700%, and 5.800% of all the nonTransmembrane protein subcellular locations.

### GO enrichment analysis

Here, we found that 1005 BP, 230 CC, and 313 MF were statistically significant in TMPs while 1633 BP, 289 CC, and 269 MF were statistically significant in nonTMPs. Each category contains 10 analysis terms with the smallest P.adjust.

**Fig. 5:**
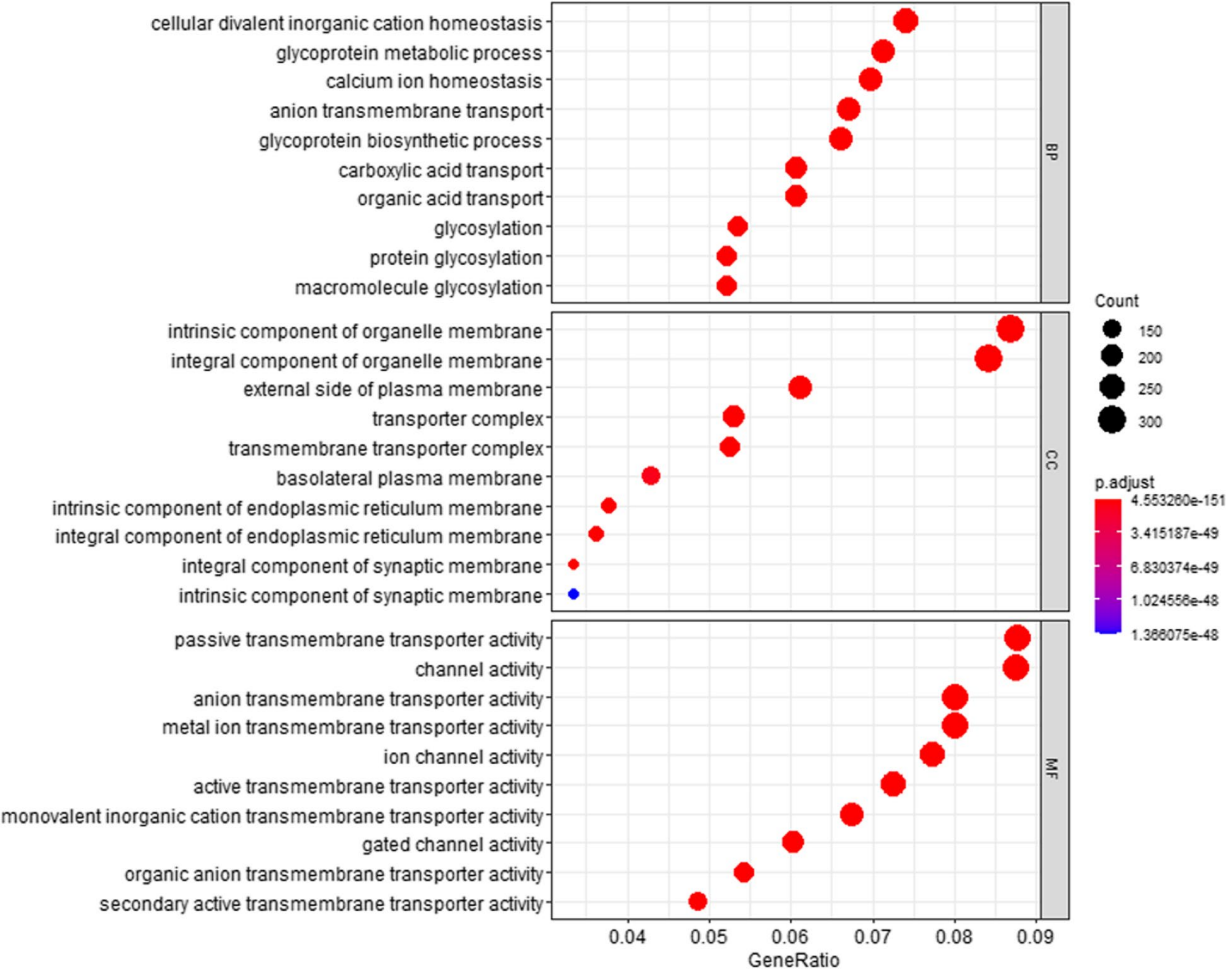
GO annotation of transmembrane proteins. This figure is used to characterize the top 10 results of the functional enrichment analysis of transmembrane proteins for each group. Dots represent term enrichment with color coding: red indicates high enrichment, blue indicates low enrichment. The sizes of the dots represent the gene ratio of each term. The larger the dot, the larger percentage of genes. For example, in the Cellular Component (CC) category, the blue point has a small gene ratio and has the least significant *P*-value compared to other terms in the figures

GO analysis of TMPs shown in Fig. 5 demonstrated that (1) for BP, anion transmembrane transport, glycoprotein biosynthetic process, glycoprotein metabolic process, protein glycosylation, macromolecule glycosylation, glycosylation, carboxylic acid transport, organic acid transport, cellular divalent inorganic cation homeostasis, and calcium ion homeostasis were the biological process in which TMPs are most involved. Proteins enriched in serveral top BP items are PSN1_HUMAN, S39A8_HUMAN, OSTB_HUMAN, and so on; (2) for CC, identified proteins were significantly enriched in integral component of organelle membrane, an intrinsic component of organelle membrane, an intrinsic component of endoplasmic reticulum membrane, an integral component of endoplasmic reticulum membrane, transmembrane transporter complex, transporter complex, an integral component of synaptic membrane, external side of the plasma membrane, basolateral plasma membrane and intrinsic component of synaptic membrane. Proteins enriched in serveral top CC items are such as PKD2_HUMAN, STX1A_HUMAN, PORCN_HUMAN and so on; (3) for molecular function MF, anion transmembrane transporter activity, active transmembrane transporter activity, passive transmembrane transporter activity, channel activity, metal ion transmembrane transporter activity, organic anion transmembrane transporter activity, ion channel activity, monovalent inorganic cation transmembrane transporter activity, secondary active transmembrane transporter activity were statistically significant. Proteins enriched in serveral top MF items are VGLU1_HUMAN, S4A11_HUMAN, CLCN3_HUMAN and so on.

**Fig. 6:**
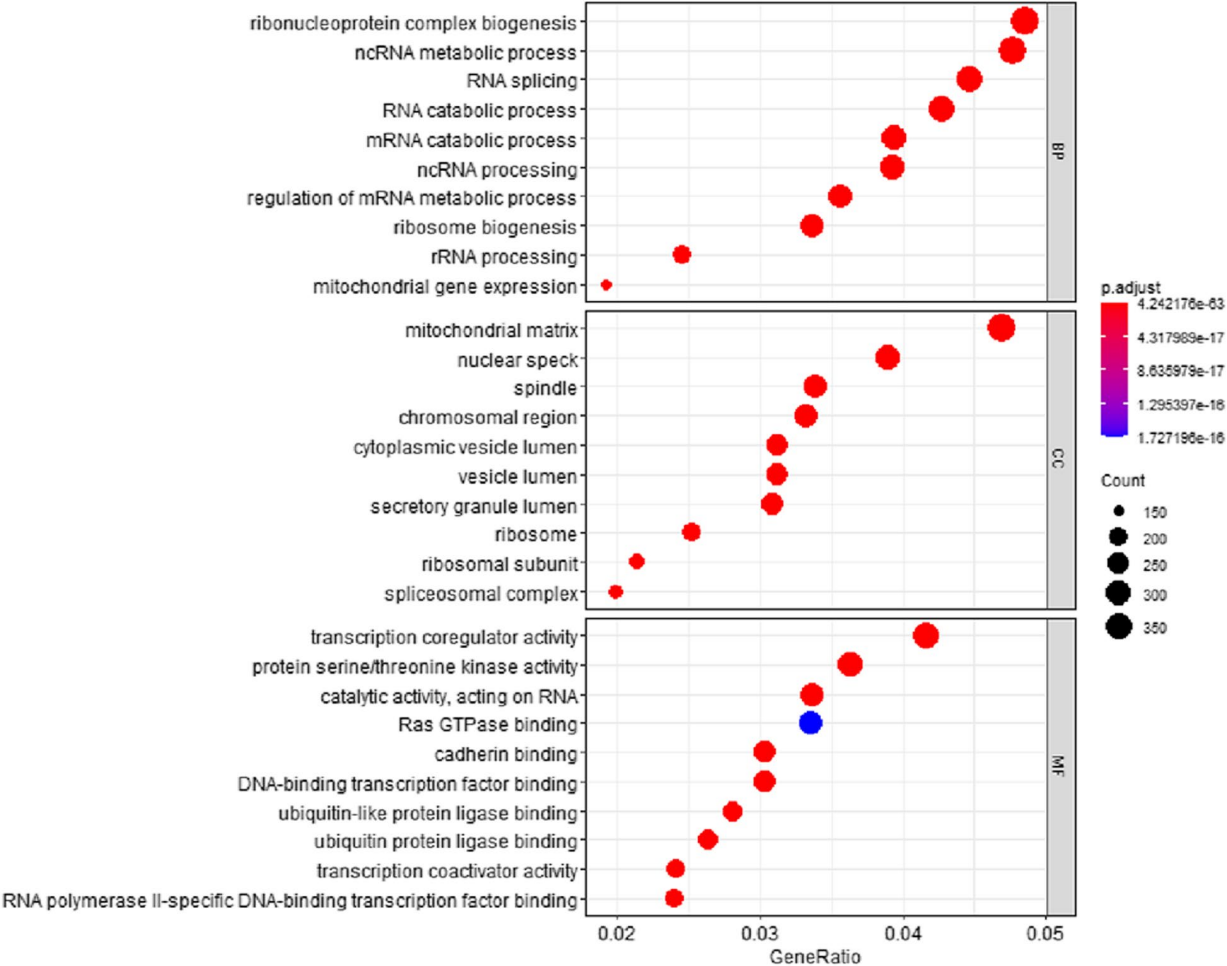
GO of non-transmembrane proteins. This figure is used to characterize the top 10 results of the functional enrichment analysis of non-transmembrane proteins for each group. Dots represent term enrichment with color coding: red indicates high enrichment, blue indicates low enrichment. The sizes of the dots represent the gene ratio of each term. The larger the dot, the larger percentage of genes. For example, Ras GTPase binding of the MF (Molecular Function) category is of least significant with the highest *P*-value compared to other terms. While ribonucleoprotein complex biogenesis in the BP(Biological Process) category accounts for more genes than others

GO analysis of nonTMPs in Fig. 6 demonstrated that (1) for BP, ten terms with the most significant enrichment in this class are listed. They are ribonucleoprotein complex biogenesis, RNA catabolic process, mRNA catabolic process, ribosome biogenesis, ncRNA metabolic process, regulation of mRNA metabolic process, ncRNA processing, RNA splicing, mitochondrial gene expression, and rRNA processing. Proteins enriched in several top BP items are MET16_HUMAN, EXOS8_HUMAN, IF4A3_HUMAN, and so on; (2) for CC, identified proteins were significantly enriched in the mitochondrial matrix, nuclear speck, ribosomal subunit, chromosomal region, secretory granule lumen, cytoplasmic vesicle lumen, vesicle lumen, spindle, and spliceosomal complex. Proteins enriched in serveral top CC items are MK14_HUMAN, PPIE_HUMAN, DDX3X_HUMAN, and so on; (3) for MF, cadherin binding, transcription coregulator activity, catalytic activity, acting on RNA, DNA-binding transcription factor binding, ubiquitin-like protein ligase binding, protein serine/threonine kinase activity, transcription coactivator activity, ubiquitin-protein ligase binding, RNA polymerase II-specific DNA-binding transcription factor binding, Ras GTPase binding was highly associated with nonTMPs. Proteins enriched in serveral top MF items are BCL10_HUMAN, PKN1_HUMAN, ARRB1_HUMAN, and so on.

### KEGG pathway enrichment analysis

**Fig. 7:**
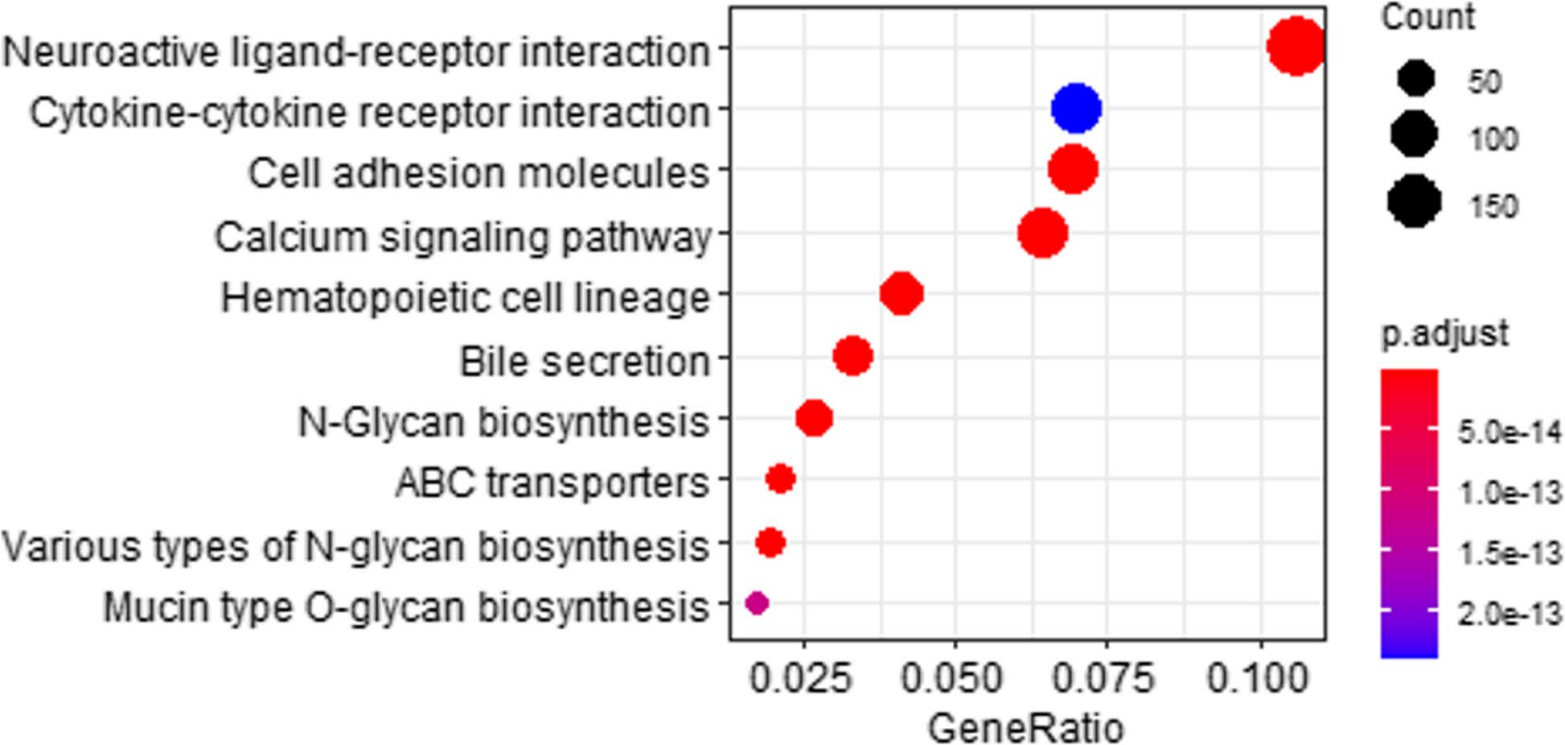
KEGG pathway enrichment of transmembrane proteins. This figure shows the top 10 results of the KEGG pathway enrichment analysis of transmembrane proteins. Dots represent term enrichment with color coding: red indicates high enrichment, blue indicates low enrichment. The sizes of the dots represent the gene ratio of each term. The larger the dot, the larger percentage of genes

By analyzing the KEGG pathway enrichment of the proteins, we found that 84 pathways were statistically significant for TMPs and 163 pathways were statistically significant for nonTMPs. All the protein mentioned are listed in Table 1, which shows protein name and simple discriptions in UniProt. KEGG pathway analysis demonstrated that TMPs (shown in Fig. 7) were particularly enriched in cell adhesion molecules, signaling pathways, biosynthesis, transport, and receptor pathways.

**Table 1:** Protein details in enriched KEGG PATHWAY

Accession ID	Name	Description
P01730	CD4_HUMAN	T-cell surface glycoprotein CD4
P28068	DMB_HUMAN	HLA class II histocompatibility antigen, DM beta chain
P04440	DPB1_HUMAN	HLA class II histocompatibility antigen, DP beta 1 chain
P23229	ITA6_HUMAN	Integrin alpha-6
Q30154	DRB5_HUMAN	HLA class II histocompatibility antigen, DR beta 5 chain
P20036	DPA1_HUMAN	HLA class II histocompatibility antigen, DP alpha 1 chain
P20273	CD22_HUMAN	B-cell receptor CD22
P01732	CD8A_HUMAN	T-cell surface glycoprotein CD8 alpha chain
P11215	ITAM_HUMAN	Integrin alpha-M
P06340	DOA_HUMAN	HLA class II histocompatibility antigen, DO alpha chain
O00329	PK3CD_HUMAN	Phosphatidylinositol 4,5-bisphosphate 3-kinase catalytic subunit delta isoform
Q9Y4K3	TRAF6_HUMAN	TNF receptor-associated factor 6
P61586	RHOA_HUMAN	Transforming protein RhoA
P60953	CDC42_HUMAN	Cell division control protein 42 homolog
P42338	PK3CB_HUMAN	Phosphatidylinositol 4,5-bisphosphate 3-kinase catalytic subunit beta isoform
P42336	PK3CA_HUMAN	Phosphatidylinositol 4,5-bisphosphate 3-kinase catalytic subunit alpha isoform
O15511	ARPC5_HUMAN	Actin-related protein 2/3 complex subunit 5
Q9P1U1	ARP3B_HUMAN	Actin-related protein 3B · Homo sapiens (Human)
Q9Y6K9	NEMO_HUMAN	NF-kappa-B essential modulator
Q92569	P55G_HUMAN	Phosphatidylinositol 3-kinase regulatory subunit gamma

**Fig. 8:**
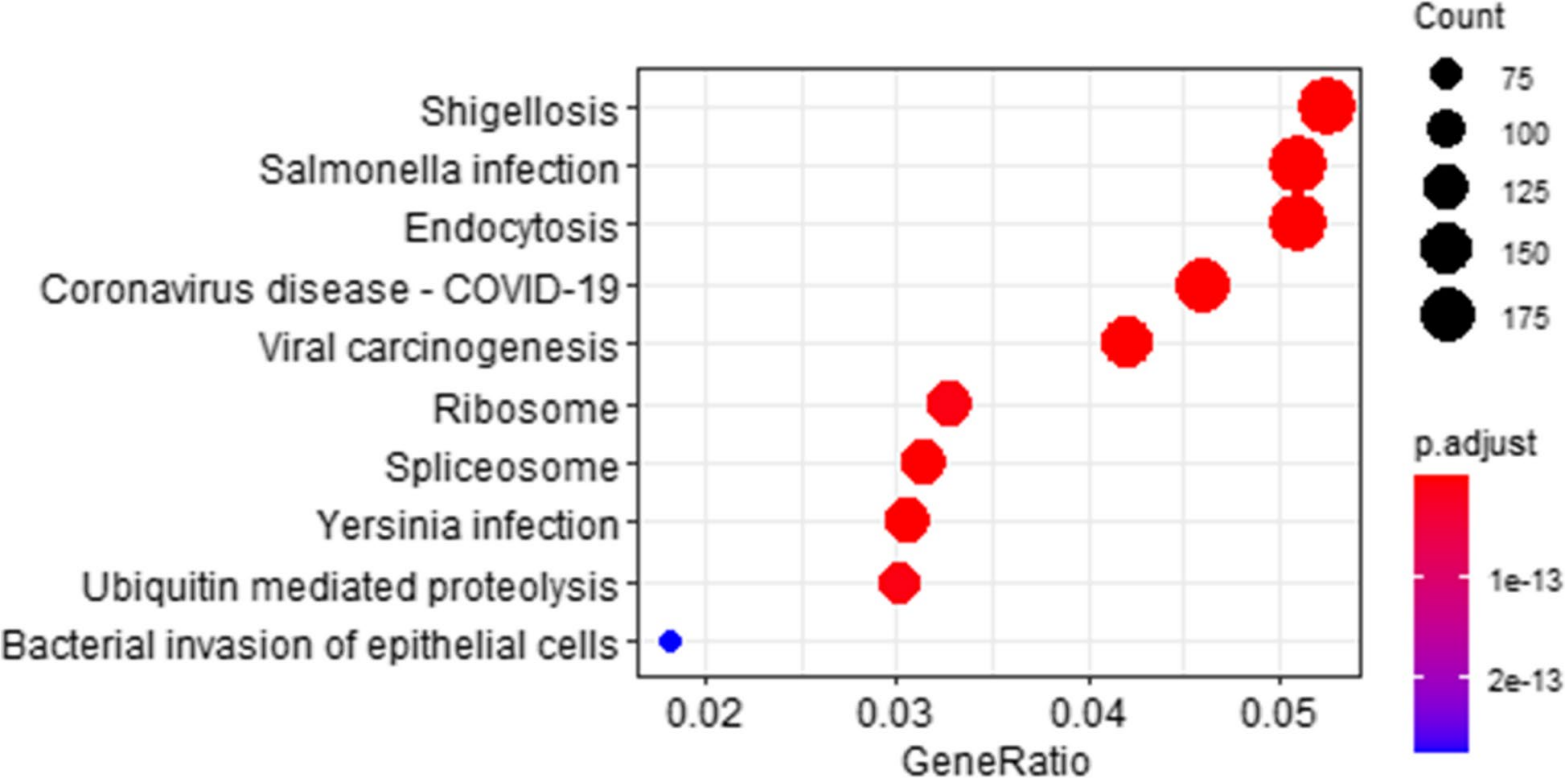
KEGG pathway enrichment of non-transmembrane proteins. This figure shows the top 10 results of the KEGG pathway enrichment analysis of non-transmembrane proteins. The y-axis was the name of signaling pathways and the x-axis was the gene ratio. Dots represent term enrichment with color coding: red indicates high enrichment, blue indicates low enrichment. The sizes of the dots represent the gene ratio of each term. The larger the dot, the larger percentage of genes

The nonTMPs (shown in Fig. 8) were particularly enriched in infection, disease, and protein-making-related pathways. Some nonTMPs such as O00329, Q9Y4K3, P61586, P60953, P42338, P42336, O15511, Q9P1U1, and Q9Y6K9 simultaneously appeared in shigellosis and salmonella infection pathways. Especially, nonTMPs such as O00329, Q9Y4K3, P42338, P42336, Q9Y6K9, Q92569 simultaneously appeared in coronavirus disease - COVID-19 pathways.

### PPI network construction and analysis

**Fig. 9:**
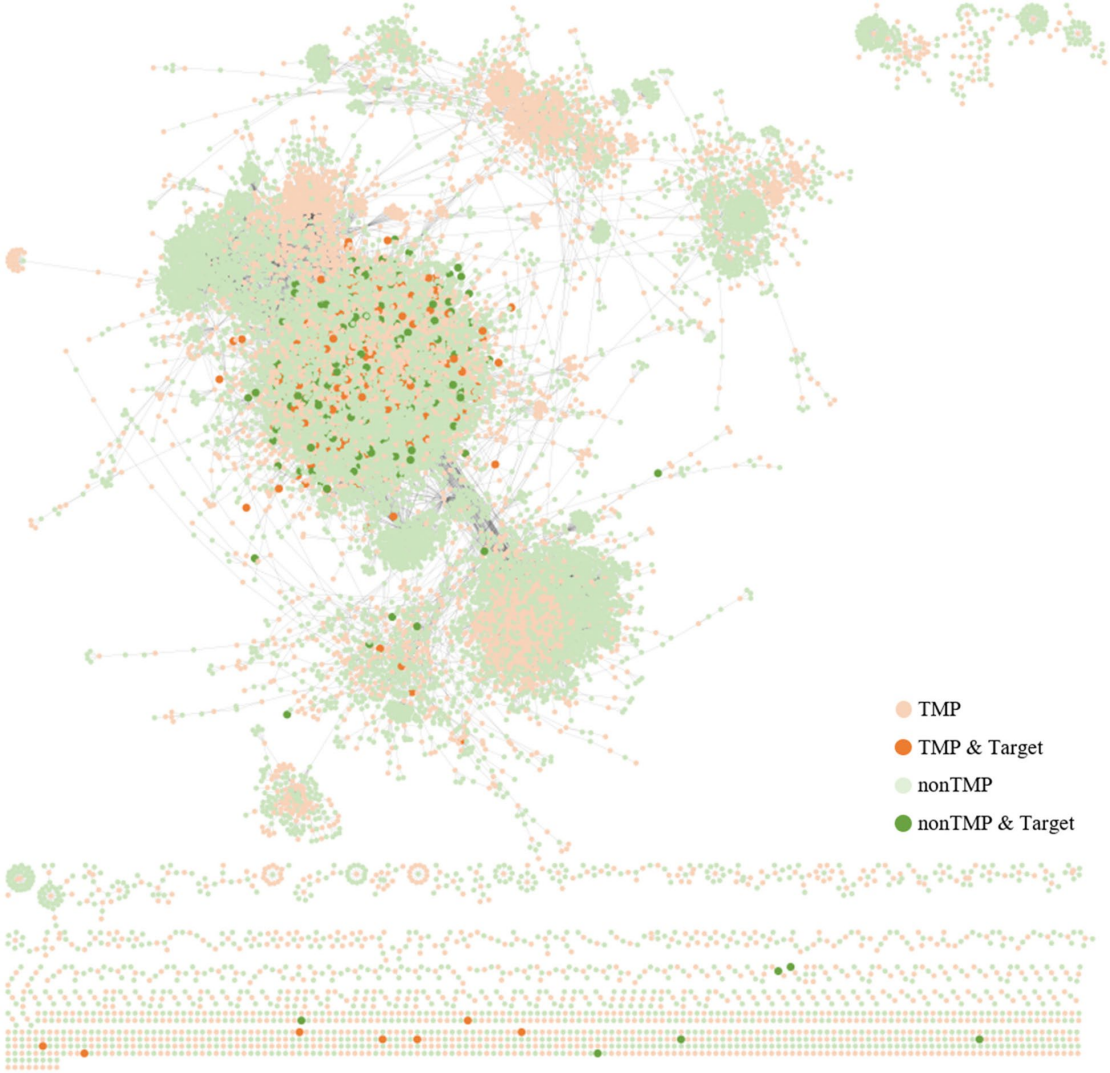
Network visualized on TMP-nonTMP interactions in POSI. Proteins were presented by nodes and the interactions between them were presented by edges. The nodes in orange were TMPs while the nodes in green were nonTMPs. Besides, Proteins recorded in the DrugBank were represented by dark orange or dark green nodes separately for TMP or nonTMPs. The number of lines connected to the nodes represented the degree of the node

The PPIs in the *POSI* were visualized by Cytoscape software [61]. From the whole network shown in Fig. 9, we can see most of the drug targets are distributed in the center of the whole network and they are very sparse existed. There remains space for researchers to find a new target from existing PPI interactions.

**Fig. 10:**
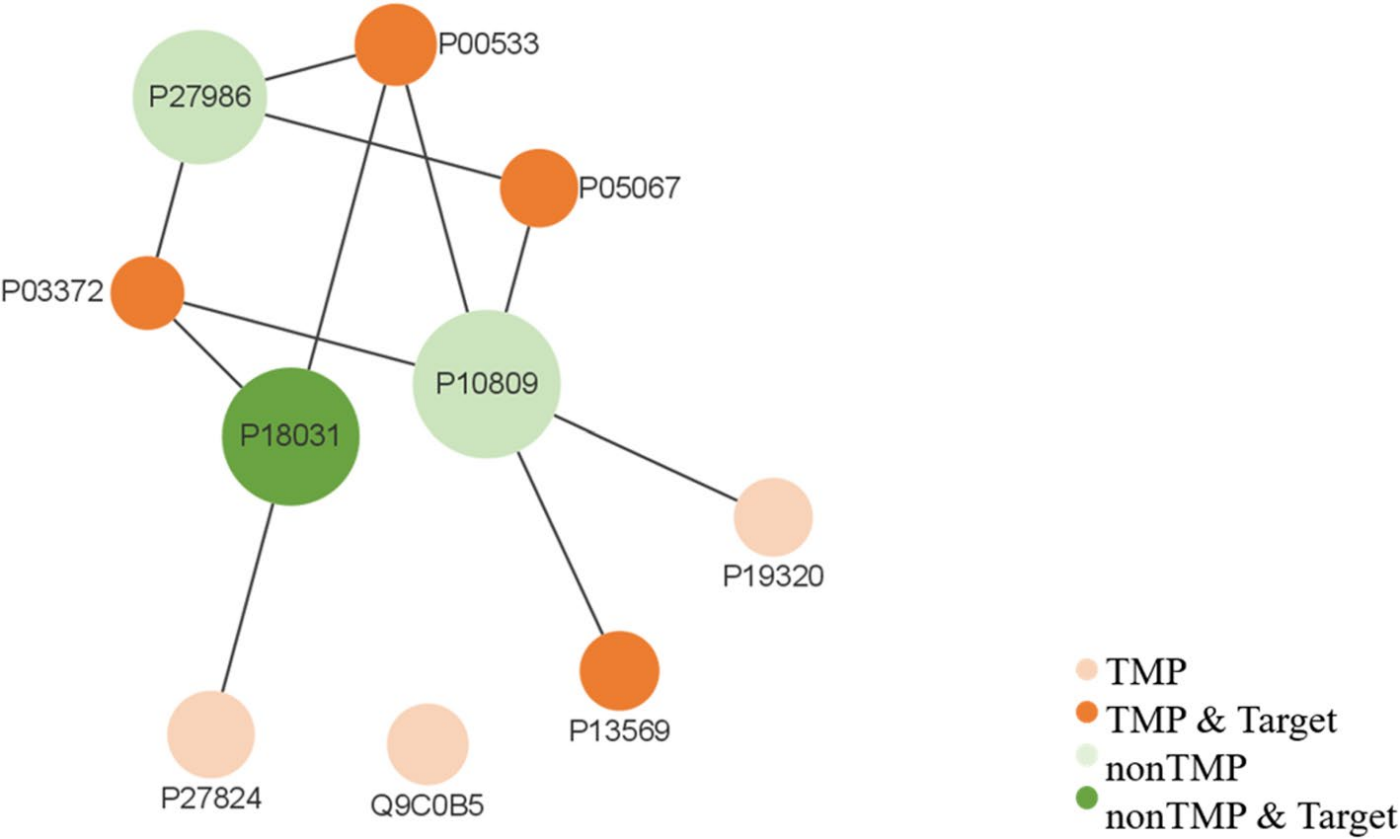
Top 10 hub proteins from POSI calculated by Closeness algorithm. Proteins are presented by nodes and the interactions between them are presented by edges. A bigger node indicates a more important protein in the dataset of POSI. The nodes in orange were TMPs while the nodes in green were nonTMPs. Besides, Proteins recorded in the DrugBank were represented by dark orange or darg green nodes separately for TMP or nonTMPs. The number of lines connected to the nodes represented the degree of the node

Furthermore, to accurately identify the hub proteins of the PPI network, cytoHubba, a plugin of Cytoscape, was adopted to identify the important nodes in the network [62]. The top 10 important proteins were kept. As is shown in Fig. 10, the size of the nodes was determined by the value of importance which was calculated by Closeness. It ranks the nodes based on the shortest paths. All the important proteins were interacting with each other except Q9C0B5. Among the top 10 important proteins, there existed a nonTMP drug target P18031 (Tyrosine-protein phosphatase non-receptor type 1). There are 4 TMP drug targets (P00533, P03372, P13569, P05067) among the top 10 important.

**Fig. 11:**
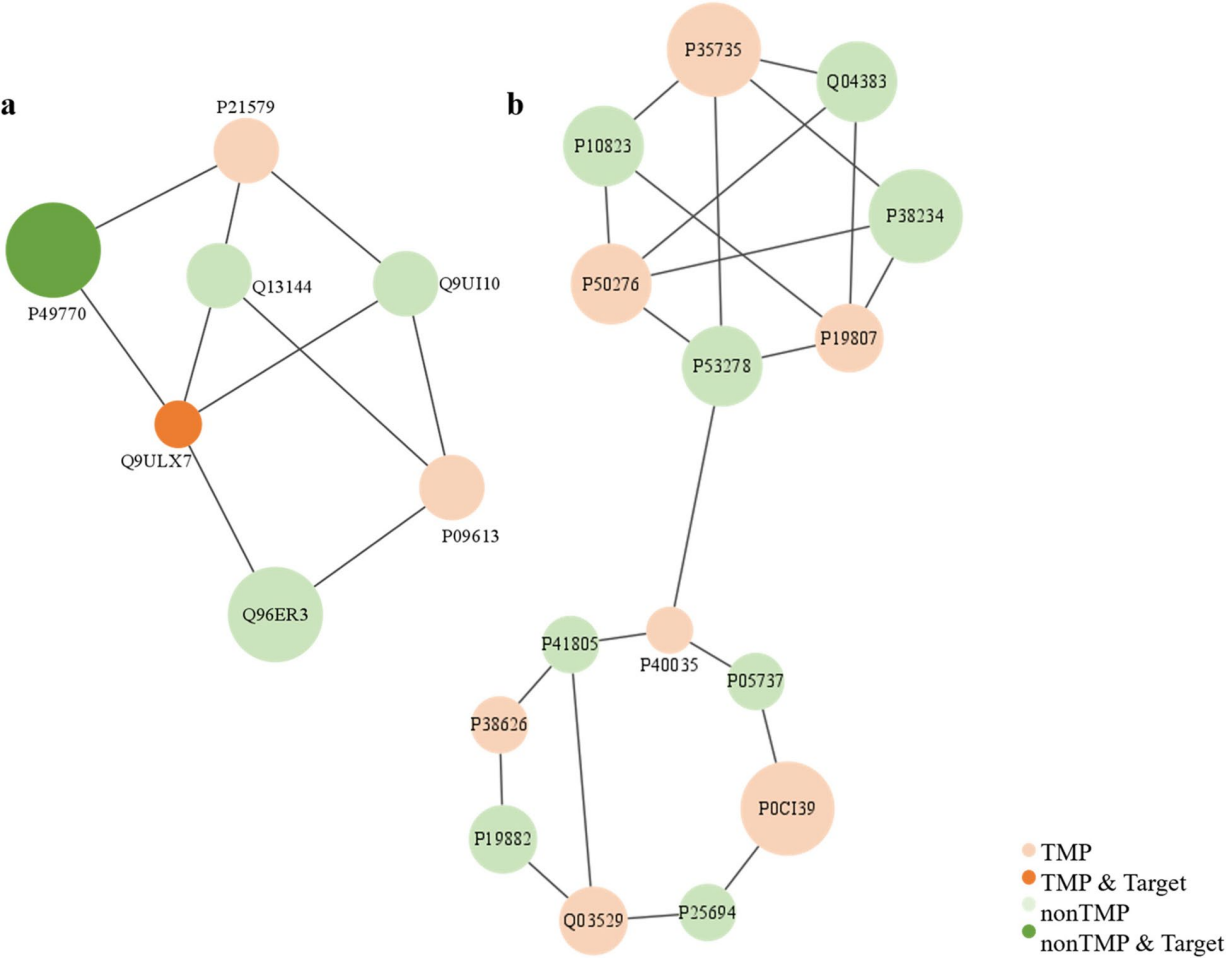
Top 2 subnetwork topologies from POSI calculated by MCODE algorithm. **a** The first cluster found by MCODE, reflected the recycling effect of the proteins. **b** The second cluster fund by MCODE reflected a bridge to connect two sub-clusters. Proteins are presented by nodes and the interactions between them are presented by edges. A bigger node indicates a more important protein in the topology of the cluster. The nodes in orange were TMPs while the nodes in green were nonTMPs. Besides, Proteins recorded in the DrugBank were represented by dark orange or darg green nodes separately for TMP or nonTMPs. The number of lines connected to the nodes represented the degree of the node

To find protein complexes and criterial parts of biological pathways in large protein interaction networks, we identified protein modules in the PPI network with Molecular Complex Detection (MCODE) algorithm [63]. It detects densely connected regions based on topology from a given network PPI networks. Those densely connected regions, also known as clusters and protein modules, may represent protein complexes or part of biological pathways. Protein modules in the PPI network were identified by the MCODE app (a plugin that implements the MCODE algorithm) in Cytoscape. Totally 36 clusters were identified as the most significant by MCODE with degree cutoff = 2, node score cutoff = 0.2, k-core = 2, and Max depth = 100. Figure 11 displays the two most important clusters, and nodes are colored to denote TMP, nonTMP, and drug target. The size of the node is decided by the value of the node score calculated by MCODE. In the first cluster, Q9ULX7 (Carbonic anhydrase 14), a TMP drug target, is the most important protein in the subnetwork. In the second cluster, there is no protein was labeled as a drug target, exploring drug targets from this group of proteins may achieve a good result.

### Performance of the predictor

To avoid contingency, we trained and tested the model with each group of datasets separately, using the average value of five models as the final performance. Images of six evaluation indexes varied with epoch increasing were plotted in (details illustrated in Additional file 1) to show the models were trained to converge on each index. Table 2 shows that the values of the five evaluation indexes have only a small range of changes, and the average Recall is 0.804, MCC 0.541, which means that the prediction results of the neural network model constructed in this study have achieved good results.

**Table 2:** Performance on the testing set of *BENCH* datasets

**Subset**	**Acc**	**Precision**	**Recall**	**F1score**	**MCC**
0	**0.790**	0.770	0.813	**0.789**	**0.581**
1	0.788	**0.828**	0.721	0.769	0.578
2	0.758	0.705	**0.894**	0.786	0.537
3	0.752	0.733	0.781	0.755	0.503
4	0.750	0.729	0.811	0.766	0.503
mean	0.768	0.753	0.804	0.773	0.541

## Discussion

We performed a comprehensive statistical analysis of known TMP-nonTMP interactions and constructed a deep learning-based predictor to identify potential interactions. The study accomplishes key links in the protein network, which facilitates the understanding of protein function and contributes to the study of disease pathogenesis and the development of related drugs. Analysis of the distribution of proteins provides a new perspective to understand the TMP-nonTMP interactions which were experimentally identified pairs and collected from a wide range of literature by IntAct. Counting the top 10 frequently occurring items for protein species and protein families, we found that the TMPs and nonTMPs share three identical high-frequency species, like Human, Yeast, and Mouse. That means, most of the proteins were belonging to the human category followed by yeast and mouse while the rest species occupied a very low part. Interactions established in the same species urgently need attention. Researches care more about what interactions is occurred in living organisms, especially in the human body.

In TMPs, as is shown in Fig. 3a, frequently occurring families were 7tm_1 (7 transmembrane receptors), PK_Tyr_Ser-Thr (Protein tyrosine and serine/threonine kinase), LRR_8 (Leucine rich repeat), and so on. 7tm_1 known as G-protein-coupled receptors, or GPCRs, are integral membrane proteins that contain seven membrane-spanning helices, and 7tm_1 are the target of around half of all modern medicinal drugs. Their expression on the cell surface makes them readily accessible to hydrophilic drugs and their non-uniform expression provides selectivity in activating or blocking physiological events. PK_Tyr_Ser-Thr which are the high-affinity cell surface receptors for many polypeptide growth factors, cytokines, and hormones catalyzes the transfer of a phosphoryl. PK_Tyr_Ser-Thr have been shown not only to be key regulators of normal cellular processes but also to have a critical role in the development and progression of many types of cancer. LRR_8 having been identified in a large number of functionally unrelated proteins contains a set of horseshoe fold proteins, closely related to protein’s structures. And they are frequently involved in the formation of PPI. In nonTMPs, as is shown in Fig. 3b, frequently occurred families were Pkinase (Protein kinase domain), zf-C2H2 (Zinc finger, C2H2 type), WD40 (WD40 repeat). Pkinase is a structurally conserved protein domain bringing a conformational change to affect the catalytic function of protein kinases. This functions as an on/off switch for many cellular processes, including metabolism, transcription, cell cycle progression, cytoskeletal rearrangement and cell movement, apoptosis, and differentiation. They also function in embryonic development, physiological responses, and the nervous and immune system. The zinc finger is the coordination of zinc ions and the C2H2 type is the best-characterized class of zinc fingers and they play important roles in cellular processes such as development, differentiation, and oncosuppression. WD40 is a short structural motif of approximately 40 amino acids being implicated in a variety of functions ranging from signal transduction and transcription regulation to cell cycle control, autophagy, and apoptosis. The subcellular locations of proteins are closely related to their function and constitute an essential aspect of understanding the complex machinery of living cells [64].

### Pathways closely related to the interaction between TMP-non TMP

KEGG pathway analysis demonstrated that TMPs were particularly enriched in cell adhesion molecules, signaling pathways, biosynthesis, transport, and receptor pathways. TMP P01730 simultaneously appeared in cell adhesion molecules, hematopoietic cell lineage, and cytokine-cytokine receptor interaction. Some TMPs such as P28068 , P04440, P23229, Q30154, P20036, P20273, P01732, P11215, and P06340 simultaneously appeared in cell adhesion molecules and hematopoietic cell lineage pathways. Cell adhesion molecules (CAMs) are a group of transmembrane proteins that are associated with neurite formation and axon pathfinding during circuitry development [65]. There are a lot of diseases associated with it, such as epidermolysis bullosa [66], ectodermal dysplasia [67], macular dystrophy [68], and neonatal ichthyosis-sclerosing cholangitis (NISCH) syndrome [69]. Hematopoietic stem cells (HSCs) are multipotent, self-renewing progenitor cells from which all differentiated blood cell types arise during the process of hematopoiesis. Cells undergoing the differentiation process express a stage- and lineage-specific set of surface markers. And These cells become diseased and can lead to Hemophilia [70], Bernard-Soulier syndrome [71], Castleman [72] and such disease like that. Via pathways, proteins can act as a biomarker to help diagnosed diseases, significantly help increase the chances of cure [73]. The nonTMPs were particularly enriched in infection, disease, and protein-making-related pathways. Some nonTMPs such as O00329, Q9Y4K3, P61586, P60953, P42338, P42336, O15511, Q9P1U1, and Q9Y6K9 simultaneously appeared in shigellosis and salmonella infection pathways. Especially, nonTMPs such as O00329, Q9Y4K3, P42338, P42336, Q9Y6K9, Q92569 simultaneously appeared in coronavirus disease - COVID-19 pathways. These annotations will be immediately useful for identifying additional relevant interacting proteins, assessing possible effects of variation in the host or viral proteins on specific steps of viral infection, and identifying possible drug targets. Thus, nonTMP that interact with TMPs are closely related to disease-related pathways and we can explore potential drugs from those candidates in the future.

### Hub genes in protein-protein interaction network

The PPIs in the *POSI* were visualized by Cytoscape software [61]. From the whole network, we can see that some proteins, forming a dense network, were fully researched because there are so many interactions documented in the literature. However, some proteins are lonely exists due to their interaction only with specific proteins or being ignored by the researchers. Most of the drug targets are distributed in the center of the whole network and they are very sparse existed. There remains space for researchers to find a new target from existing PPI interactions.

To accurately identify the hub proteins of the PPI network, cytoHubba, a plugin of Cytoscape, was adopted to identify the important nodes in the network. We found all the important proteins were interacting with each other except Q9C0B5. Among the top 10 important proteins, there existed a nonTMP drug target P18031 (Tyrosine-protein phosphatase non-receptor type 1). It may regulate the EFNA5-EPHA3 signaling pathway which modulates cell reorganization and cell-cell repulsion, and it also regulates the hepatocyte growth factor receptor signaling pathway through dephosphorylation of MET [74]. We also found there are 4 TMP drug targets (P00533, P03372, P13569, P05067) among the top 10 important. P00533 (epidermal growth factor receptor, EGFR), a human TMP with the Pkinase_Tyri family, is not only the hub of the POSI but also linked to lung cancer. This kind of protein is overexpressed in many human tumors, being recognized as a potential drug target in oncology. During SARS infections, it was found that inhibiting EGFR signaling prevents excessive fibrotic responses and, thus, lung damage. Drugs, like brigatinib, afatinib, osimertinib, and so on, were possible EGFR inhibitors. P03372 (Estrogen receptor, ESR1), a human TMP with the ESR1 family, is closely related to the disease of estrogen resistance (ESTRR) which was caused by the variants of this gene. In the case of elevated serum levels of estrogen, the disease is characterized by partial or complete resistance to estrogen. Clinical features such as osteoporosis, reduced bone mineral density, may be present.

This kind of topology of the subnetwork indicates that those groups of proteins tend to form complexes [63]. We noticed that the interaction between P40035 and P53278 are very important to bridge the two groups of proteins, this kind of interaction is documented by Krishnan et al [74].

## Conclusions

In this work, we firstly focused on TMP-nonTMP interactions and comprehensively analyzed them using statistical methods based on biological knowledge. By analyzing the distribution of the interaction pairs from several views, we found that 25.7% of the interactions took place at the cell membrane, endoplasmic reticulum membrane. The top three protein families of TMPs were 7tm_1, PK_Tyr_Ser-Thr, LRR_8 while the top three protein families of nonTMPs were Pkinase, C2H2, WD40. 73.5% of all the drug targets were closely related to the candidates of the interaction pairs. By analyzing the GO enrichment of the proteins, we found that 1005 BP, 230 CC, and 313 MF were statistically significant in TMPs while 1633 BP, 289 CC, and 269 MF were statistically significant in nonTMPs. By analyzing the KEGG pathway enrichment of the proteins, we found that 84 pathways were statistically significant for TMPs. Here, P01730 simultaneously appeared in cell adhesion molecules, hematopoietic cell lineage, and cytokine-cytokine receptor interaction. 163 pathways were found to be statistically significant for nonTMPs. Finally, characteristics of the network constructed by the interaction pairs were fully explored, showing that 10 proteins such as P00533, P03372, and P13569 are hub proteins. Five of them are drug targets, and P18031 is the most critical drug target of Ertiprotafib and Trodusquemine. And it is known to be a signaling molecule that regulates a variety of cellular processes including cell growth, differentiation, mitotic cycle, and oncogenic transformation. Furthermore, we also found the top 7 critical sub-networks. General protein-protein interaction predictors that depend on a large proportion of soluble protein pairs are not suitable to predict the sparse TMP-nonTMP interactions. Finally, characteristics of the network constructed by the interaction pairs were fully explored, finding the top 10 hub proteins and top 7 critical sub-networks.

General protein-protein interaction predictors that depend on a large proportion of soluble protein pairs are not suitable to predict the sparse TMP-nonTMP interactions. We proposed a deep learning-based prediction method called SeqTMPPI to solve the problem which is not suitable to predict the sparse TMP-nonTMP interactions. Our prediction method achieved an MCC of 0.541 over the testing set of the benchmark dataset. We were the first to provide the predictor of TMP-nonTMP interacting pairs. The study of TMP PPIs will be promisingly beneficial to understanding TMPs’ functions, completing the PPI network, and discovering potential drug targets.

### Supplementary Information

**Supplementary Material 1.**

## Data Availability

Materials and code related are available at https://github.com/NENUBioCompute/SeqTMPPI.

## Abbreviations

TMPs: Transmembrane proteins
nonTMPs: non-transmembrane proteins
CNN: Convolutional Neural Network
PPIs: Protein-protein interactions
PSSM: Position Specific Score Matrix
MCC: Matthews Correlation Coefficient
GO: Go Ontology
Pfam: Protein family
TTD: Therapeutic Target Database
GPCRs: G-Protein-Coupled Receptors
G protein: Guanine Nucleotide-Binding Protein
KEGG: Kyoto Encyclopedia of Gene and Genome
BP: Biological Process
CC: Cellular Component
MF: Molecular Function
GAP: Global Average Pooling
Acc: Accuracy
TP: True Positive
TN: True Negative
FP: False Negative
FN: False Positive
