# Supplementary material for: Analysis and prediction of interactions between transmembrane and non-transmembrane proteins
Source: BMC Genomics. 2024 Apr 24;25(Suppl 1):401. doi: 10.1186/s12864-024-10251-z (PMC11040819; doi:10.1186/s12864-024-10251-z)
Supplement: Supplementary file 1 — Supplementary Material 1. [file 12864_2024_10251_MOESM1_ESM.docx]

Analysis and Prediction of Interactions between Transmembrane and Non-transmembrane Protein

Chang Lu^1^, Jiuhong Jiang^1^, Qiufen Chen^1^, Huanhuan Liu^1^, Xingda Ju^1^, Han Wang^1,*^

^1^ School of Psychology, School of Information Science and Technology, Institute of Computational Biology, Northeast Normal University, Changchun, China.

^*^ Correspondence: wangh101@nenu.edu.cn.

# Supplementary material

The PDF file includes:

Supplemental Text

Tables S1~S4

Figs. S1~S7

Datasets, models, and code sources for this manuscript are included in the following:

(available at <https://github.com/NENUBioCompute/SeqTMPPI/>.)

1. **Details of the Benchmark dataset**

**Table S1.** Detailed statistics of the samples in *BENCH*

| **Dataset** | **Type** | | **Positive** | **Negative** | **Sum** |
| --- | --- | --- | --- | --- | --- |
| *BENCH* | train | | 52056 | 51846 | 103902 |
|  | validate | | 6370 | 6617 | 12987 |
|  | test | | 6511 | 6476 | 12987 |
|  | all | | 64937 | 64939 | 129876 |
| 5 subsets | 0 | train | 10426 | 10353 | 20779 |
|  |  | validate | 1358 | 1239 | 2597 |
|  |  | test | 1321 | 1276 | 2597 |
|  |  | all | 12987 | 12987 | 25974 |
|  | 1 | train | 10399 | 10380 | 20779 |
|  |  | validate | 1299 | 1298 | 2597 |
|  |  | test | 1309 | 1288 | 2597 |
|  |  | all | 12987 | 12987 | 25974 |
|  | 2 | train | 10395 | 10384 | 20779 |
|  |  | validate | 1304 | 1293 | 2597 |
|  |  | test | 1299 | 1298 | 2597 |
|  |  | all | 12987 | 12987 | 25974 |
|  | 3 | train | 10423 | 10356 | 20779 |
|  |  | validate | 1343 | 1254 | 2597 |
|  |  | test | 1310 | 1287 | 2597 |
|  |  | all | 12987 | 12987 | 25974 |
|  | 4 | train | 10392 | 10387 | 20779 |
|  |  | validate | 1325 | 1272 | 2597 |
|  |  | test | 1323 | 1274 | 2597 |

1. **Details of Max length**

The max length of the protein is set to 2000, shown in **Fig. S1**. Among 27,962 unique proteins, there were only 740 (~3%) proteins whose lengths were longer than 2,000. Proteins with length <2000 have covered most of the situations. The interacting domain is of vital importance for the PPIs, so we think it is not a good idea to cut the sequence into several parts. Above all, the max length of the protein was set to 2,000


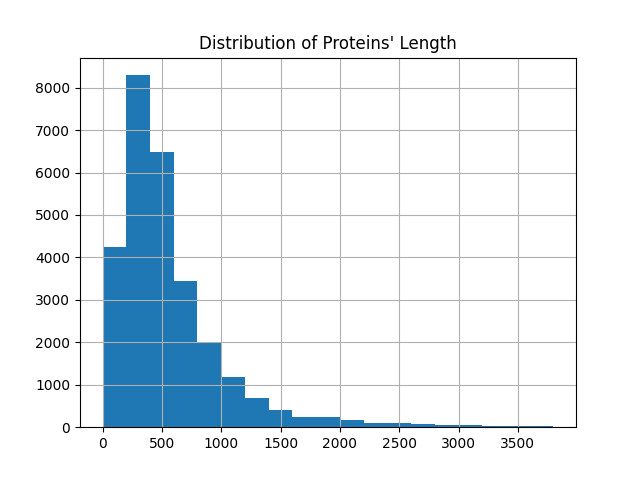


**Fig. S1.** Distribution of protein’s length

1. **Hyper-Parameter Tuning**

In the first training phase, we trained the model on the dataset *MINI*. We divided each group of the dataset into the training set, validating set, and testing set in the ratio of 8:1:1. Since there are 5 groups of samples in the dataset, we trained the network 5 times and take the average results as our performance. Results are shown in **Table S2**, we got a satisfied MCC value of 0.630, and different group of the dataset remains similar performance. We noticed that performance of group 1 was significantly better than other groups, which remains to be further explored. Initial param: kernel size = 21, filter num = 250, batch size = 150, epoch = 80.

**Table S2.** Result in different groups of dataset *MINI*.

| Subset | Loss | Acc | Pre | Recall | F_1_- score | MCC |
| --- | --- | --- | --- | --- | --- | --- |
| 1 | 0.431 | 0.790 | 0.828 | 0.736 | 0.778 | 0.583 |
| 2 | 0.429 | 0.817 | 0.844 | 0.773 | 0.807 | 0.634 |
| 3 | 0.386 | 0.826 | 0.846 | 0.845 | 0.843 | 0.655 |
| 4 | 0.469 | 0.819 | 0.812 | 0.835 | 0.822 | 0.638 |
| 5 | 0.466 | 0.819 | 0.832 | 0.779 | 0.804 | 0.639 |
| mean | 0.436±0.03 | 0.814±0.013 | 0.832±0.012 | 0.793±0.041 | 0.811±0.021 | 0.630±0.024 |

In the second training phase, we tuned the major three hyper-parameters in the model, including kernel size, the filter number, and batch size. Other parameters are fixed each time, and one of the hyperparameters is adjusted to find the parameter combination that makes the model perform best. The final hyper-parameters of this model are settled with kernel size 90, filters 300, batch size 70.

### Kernel size tuning

It defines the length of the scanner, the param often has a strong impact on Deep Learning models. We increased the kernel size by 9 each time and got a list of kernels. For each kernel, we trained the network 5 times and take the average results as our performance. **Table S2** shows the performance of models with those kernels. When kernel size is equal to 90, we got the minimum value of Loss, maximum Acc, F1score, and MCC.

**Table S2.** Performance when vary kernel size (filters = 250, batch size = 150)

| Kernel size | Loss | Acc | Precision | Recall | F1score | MCC |
| --- | --- | --- | --- | --- | --- | --- |
| 9 | 0.362 | 0.848 | 0.878 | 0.818 | 0.845 | 0.698 |
| 18 | 0.359 | 0.836 | 0.867 | 0.813 | 0.839 | 0.675 |
| 27 | 0.361 | 0.836 | 0.869 | 0.810 | 0.838 | 0.676 |
| 36 | 0.370 | 0.846 | 0.848 | 0.860 | 0.854 | 0.691 |
| 45 | 0.364 | 0.846 | 0.860 | 0.845 | 0.852 | 0.691 |
| 54 | 0.363 | 0.836 | 0.866 | 0.813 | 0.839 | 0.674 |
| 63 | 0.360 | 0.836 | 0.885 | 0.791 | 0.835 | 0.678 |
| 72 | 0.354 | 0.851 | 0.882 | 0.827 | 0.853 | 0.704 |
| 81 | 0.369 | 0.846 | 0.842 | 0.868 | 0.854 | 0.693 |
| **90** | **0.352** | **0.853** | 0.879 | 0.838 | **0.857** | **0.708** |
| 99 | 0.352 | 0.848 | **0.889** | 0.814 | 0.849 | 0.701 |
| 108 | 0.357 | 0.851 | 0.839 | **0.876** | 0.855 | 0.700 |
| 117 | 0.353 | 0.841 | 0.859 | 0.830 | 0.845 | 0.682 |

### number of filters tuning

A similar process to tuning kernel size, we increased the batch size by 30 from 60 to 210 and fixed other parameters. **Table S3** shows the performance for models with those params.

**Table S3.** Performance when vary with different filters (kernel size = 90, batch size = 150)

| Filters | Loss | Acc | Precision | Recall | F1score | MCC |
| --- | --- | --- | --- | --- | --- | --- |
| 50 | 0.364 | 0.841 | 0.862 | 0.825 | 0.842 | 0.684 |
| 100 | 0.358 | 0.846 | 0.862 | 0.842 | 0.851 | 0.692 |
| 150 | 0.356 | 0.844 | 0.871 | 0.819 | 0.844 | 0.687 |
| 200 | 0.352 | 0.846 | 0.873 | 0.829 | 0.849 | 0.696 |
| 250 | 0.358 | 0.841 | 0.855 | 0.838 | 0.846 | 0.682 |
| 300 | 0.355 | 0.853 | 0.853 | 0.875 | 0.862 | 0.707 |
| 350 | 0.359 | 0.851 | 0.838 | 0.882 | 0.859 | 0.699 |

### Batch size tuning

When set kernel size as 90, filters as 300, vary batch size between 60-170, the result in **Table S4** shows that the best batch size is 70 with MCC 0.722. Up to now, the final hyper-parameters of this model are settled with kernel size 90, filters 300, batch size 70.

**Table S4****.** performance when vary batch size (kernel size = 99, filters = 300)

| Batch size | Loss | Acc | Precision | Recall | F1score | MCC |
| --- | --- | --- | --- | --- | --- | --- |
| 60 | 0.333 | 0.858 | 0.888 | 0.830 | 0.857 | 0.717 |
| 70 | 0.333 | 0.861 | 0.871 | 0.859 | 0.864 | 0.722 |
| 80 | 0.338 | 0.856 | 0.877 | 0.844 | 0.858 | 0.715 |
| 90 | 0.349 | 0.851 | 0.852 | 0.866 | 0.858 | 0.701 |
| 100 | 0.358 | 0.844 | 0.906 | 0.781 | 0.838 | 0.697 |
| 110 | 0.350 | 0.844 | 0.898 | 0.791 | 0.841 | 0.692 |
| 120 | 0.356 | 0.844 | 0.868 | 0.831 | 0.849 | 0.688 |
| 130 | 0.353 | 0.839 | 0.878 | 0.803 | 0.838 | 0.683 |
| 140 | 0.348 | 0.848 | 0.862 | 0.846 | 0.854 | 0.697 |
| 150 | 0.355 | 0.844 | 0.853 | 0.845 | 0.849 | 0.687 |
| 160 | 0.353 | 0.851 | 0.902 | 0.803 | 0.850 | 0.707 |

1. **Visual training process**

Images of six evaluation indexes varied with epoch increasing were plotted in **Fig. S2~S7** to show the models were trained to converge on each index.
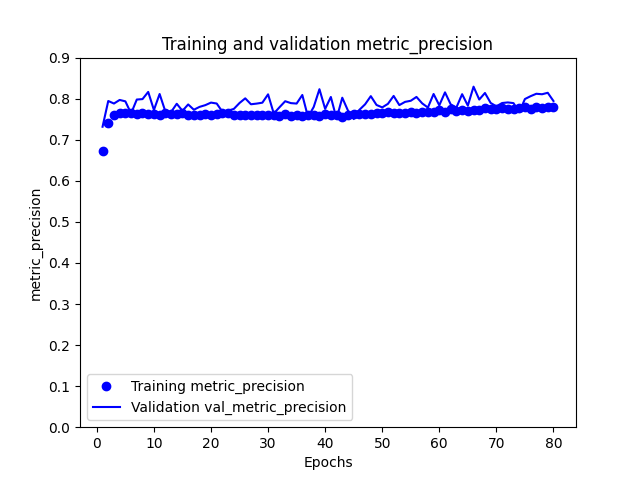


**Fig. S2**. Precision varied with epoch


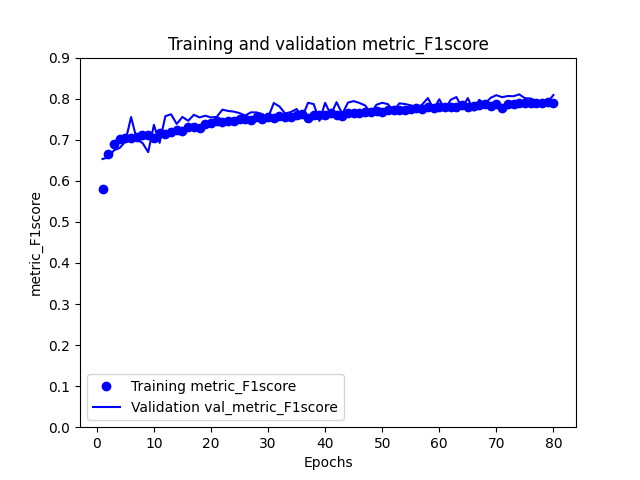


**Fig. S3.** F1-score varied with epoch


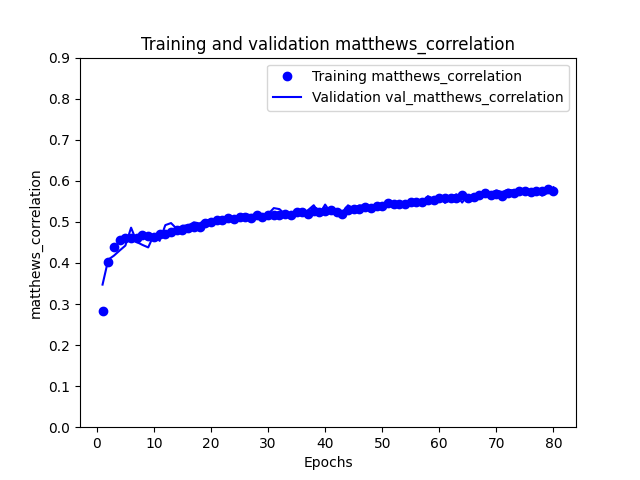


**Fig. S4.** MCC varied with epoch


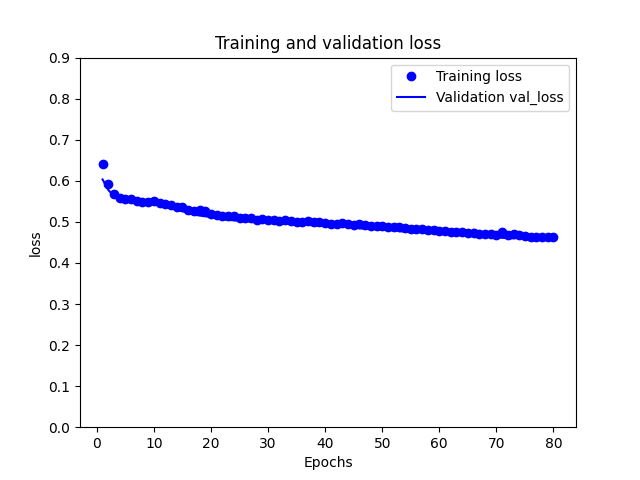


**Fig. S5.** Loss varied with epoch


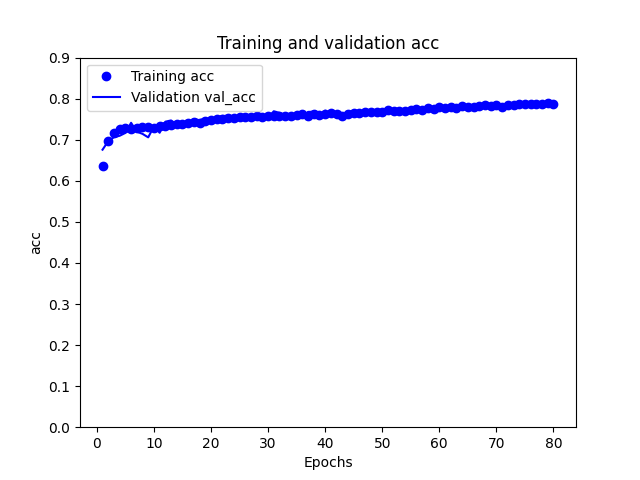


**Fig. S6.** Precision varied with epoch


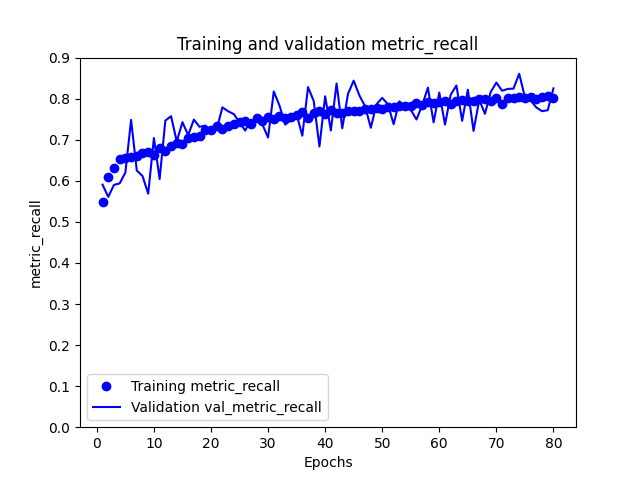


**Fig. S7.** Precision varied with epoch
